# The Cardiometabolic Risk in Women with Polycystic Ovarian Syndrome (PCOS): From Pathophysiology to Diagnosis and Treatment

**DOI:** 10.3390/medicina60101656

**Published:** 2024-10-10

**Authors:** Sotirios Pililis, Stamatios Lampsas, Aikaterini Kountouri, Loukia Pliouta, Emmanouil Korakas, Sarantis Livadas, John Thymis, Melpomeni Peppa, Sophia Kalantaridou, Evangelos Oikonomou, Ignatios Ikonomidis, Vaia Lambadiari

**Affiliations:** 1 Diabetes Center, 2nd Department of Internal Medicine, Attikon University Hospital, Medical School, National and Kapodistrian University of Athens, 12462 Athens, Greece; sotiris181@yahoo.gr (S.P.); katerinak90@hotmail.com (A.K.); mankor-th@hotmail.com (E.K.);; 2 2nd Department of Ophthalmology, Attikon Hospital, National and Kapodistrian University of Athens, 12462 Athens, Greece; 3 Endocrine Unit, Athens Medical Centre, 65403 Athens, Greece; sarantislivadas@gmail.com; 4 2nd Cardiology Department, Attikon University Hospital, National & Kapodistrian University of Athens, 12462 Athens, Greece; johnythg@gmail.com (J.T.);; 5 3rd Department of Obstetrics and Gynecology, Attikon Hospital, School of Medicine, National and Kapodistrian University of Athens, 12462 Athens, Greece; 6 3rd Department of Cardiology, Medical School, “Sotiria” Chest Diseases Hospital, National and Kapodistrian University of Athens, 11527 Athens, Greece

**Keywords:** polycystic ovarian syndrome, cardiovascular disease, metabolic syndrome, treatment, risk factors

## Abstract

Polycystic Ovarian Syndrome (PCOS) is a prevalent endocrine disorder affecting women of reproductive age, with significant variations in presentation characterized by hyperandrogenism, ovulatory dysfunction, and polycystic ovarian morphology. Beyond reproductive health, it may also pose crucial long-term cardiometabolic risks, especially for women with specific types of PCOS, contributing to early subclinical cardiovascular atherosclerotic alterations such as endothelial dysfunction, increased arterial stiffness, and coronary artery calcium levels, respectively. Moreover, the precise relationship between clinical cardiovascular disease (CVD) and PCOS remains debated, with studies demonstrating an elevated risk while others report no significant association. This review investigates the pathophysiology of PCOS, focusing on insulin resistance and its link to subclinical and clinical cardiovascular disease. Diagnostic challenges and novel management strategies, including lifestyle interventions, medications like metformin and glucagon-like peptide-1 receptor agonists (GLP-1RAs), hormonal contraceptives, and bariatric surgery, are further discussed. Recognizing the cardiometabolic risks associated with PCOS, a comprehensive approach and early intervention should address both the reproductive and cardiometabolic dimensions of the syndrome.

## 1. Introduction

Polycystic ovarian syndrome (PCOS) is among the most prevalent endocrine and metabolic disorders found in women of reproductive age, and was first described by Stein and Leventhal in 1935 [1]. The prevalence of PCOS among premenopausal women varies from ~6.6% to ~19.9 depending on the diagnostic criteria used to define the syndrome [2,3,4]. Hirsutism, acne, amenorrhea, oligomenorrhea, hyperinsulinemia, infertility, and mood disorders are some of the most common clinical symptoms of PCOS [5]. PCOS is a syndrome with multiple signs and symptoms that cannot be diagnosed by a single test, which is why there is little disagreement in the scientific community.

**Figure 1:**
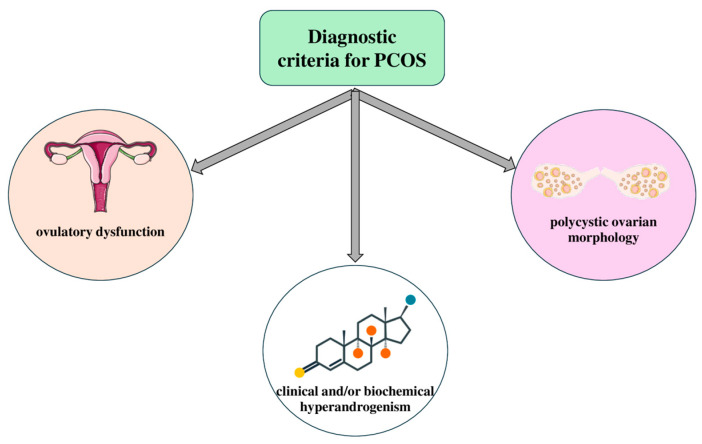
Diagnostic criteria for polycystic ovarian syndrome (PCOS). The key criteria used for diagnosing PCOS, including hyperandrogenism, ovulatory dysfunction, and polycystic ovarian morphology, based on the Rotterdam criteria and other relevant clinical guidelines. Parts of the figure were drawn using pictures from Servier Medical Art. Servier Medical Art by Servier is licensed under a Creative Commons Attribution 3.0 Unported License (https://creativecommons.org/licenses/by/3.0/ (accessed on 26 August 2024)).

The definition of PCOS is a debatable issue worldwide because of the lack of solid evidence about the etiology and pathophysiology of the disorder [6]. The accumulation of ovarian follicles at various stages of development or degeneration is one of the most characteristic features, given the name polycystic ovarian syndrome [7]. However, several names such as “hyperandrogenic–chronic anovulation”, “metabolic reproductive syndrome”, “prevalent cardiometabolic ovary syndrome” or “functional ovarian hyperandrogenism” have been proposed [8,9,10,11]. The Rotterdam criteria are the most commonly used for classifying PCOS and they are currently endorsed by the majority of scientific societies and health organizations [12]. The definition suggests that PCOS can be diagnosed in a woman if she exhibits at least two of the following three characteristics: 1. clinical and/or biochemical hyperandrogenism; 2. ovulatory dysfunction; and 3. polycystic ovarian morphology [13]. In the last 2023 International Evidence-based Guidelines, elevated anti-Mullerian hormone (AMH) levels were added as a diagnostic criterion, but it is not recommended due to overlap with the normal reproductive physiology of adolescents [14]. The diagnostic criteria are summarized in Figure 1. It is also worth noting the extensively researched phenotypic variations, reflecting differences in clinical features that can influence the severity of symptoms, metabolic risks, and treatment responses in affected individuals.

**Figure 2:**
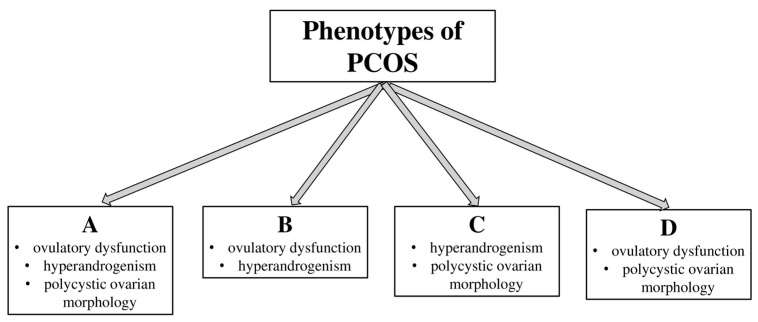
Polycystic ovarian syndrome (PCOS) phenotypes. PCOS is characterized by different variations of clinical features such as hyperandrogenism, ovulatory dysfunction, and polycystic ovarian morphology, as classified by the Rotterdam criteria.

PCOS can be categorized into four distinct phenotypes based on its presentation: 1. Phenotype A—ovulatory dysfunction (OD) and hyperandrogenism (HA) and polycystic ovarian morphology (PCOM); 2. Phenotype B—HA and OD; 3. Phenotype C—HA and PCOM; and 4. Phenotype D—OD and PCOM [15]. The prevalence of the four phenotypes of PCOS can vary depending on the population studied and the diagnostic criteria [16,17,18]. However, generally speaking, the global prevalence rates are as follows: Phenotype A—the most common phenotype, with a prevalence of about 50–60%; Phenotype B—the second most common, with a prevalence of around 20–30%; Phenotype C—the prevalence of this phenotype is approximately 10–15%; and Phenotype D—this phenotype is the least common, with a prevalence of about 5–10% [19] (Figure 2). These phenotypes highlight the variability in PCOS presentation, influencing both clinical management and long-term health risks.

Clinicians often focus on the management of specific symptoms such as ovulatory dysfunction and infertility, but it is increasingly challenging due to the syndrome’s complexity and varied presentations [20]. However, the long-term cardiometabolic risks conferred by PCOS usually remain untreated [21]. In particular, PCOS is considered a significant risk factor for cardiovascular disease (CVD), especially in patients with Phenotypes A and B, in whom hyperandrogenism, abdominal obesity, and insulin resistance are more pronounced [22]. A large Australian retrospective study of 2566 Australian subjects with PCOS reported that these women had more diagnoses of cardiovascular events, with adjusted hazard ratios (HRs) for cerebrovascular disease, ischemic heart disease, and arterial/venous disease of 2.58 (1.43–4.67), 2.89 (1.68–4.97), and 1.81 (1.59–2.05), respectively, compared to controls [23]. In addition, a meta-analysis showed a 2-fold increased risk for CVD or stroke for women with PCOS even after adjusting for several risk factors such as body mass index (BMI) when compared with healthy control subjects [24]. Moreover, a large Danish registry of 18,112 women revealed a HR 1.7 (1.7–1.8) for the development of CVD in patients with PCOS, with a total event rate of CVD of 22.6 per 1000 patient-years in patients with PCOS vs. 13.2 per 1000 patient-years in controls [25]. Although several studies demonstrate a positive relationship between PCOS and the future CVD risk [26,27,28,29], the evidence is conflicting since large meta-analyses and cohorts have also reported no statistically significant results between PCOS and the risk of CVD [26,29,30,31].

The cardiovascular risk associated with PCOS is a subject of debate in the academic community, highlighting the importance of early disease management to potentially mitigate long-term health consequences. The purpose of this article is to review reliable biomarkers that can aid in early diagnosis and provide prognostic value for the likelihood of adverse events. Also in this article, we review the emerging data for the management of these adverse events.

## 2. Cardiometabolic Risk in Patients with PCOS

### 2.1. Insulin Resistance and Hormonal Imbalance

Insulin resistance is the most common metabolic abnormality in patients with PCOS [32]. Particularly, insulin resistance and subsequent relative hyperinsulinemia are found in 65–95% of women with PCOS, affecting mostly overweight and obese women, as well as more than half of those with normal weight [33,34,35]. The underlying pathogenetic mechanism remains uncertain. Environmental factors, mitochondrial dysfunction, epigenetic alterations, and chronic inflammation seem to play a catalytic role in PCOS progression [36,37,38,39,40]. Insulin regulates glucose homeostasis by promoting glucose uptake in insulin-sensitive tissues like skeletal muscle, adipose tissue, liver, and heart, while also inhibiting glucose production in the liver [41]. In patients with PCOS, insulin resistance is characterized by a reduced ability of these peripheral tissues to respond to normal or elevated concentrations of insulin [39].

Hyperinsulinemia, a consequence of insulin resistance, stimulates ovarian androgen production by increasing the activity of theca cells, which are responsible for synthesizing androgens in the ovaries [42]. On the other hand, early androgen secretion is usually considered premature in patients with PCOS and is thought to cause insulin resistance in the earlier stages [43]. As a result, hyperandrogenism lowers the levels of sex hormone-binding globulin (SHBG), leading to an increased concentration of free testosterone [44]. Excessive exposure to androgens directly and specifically affects the development of insulin resistance [45]. Particularly, elevated testosterone levels in plasma can be converted to estrone in excess adipose tissue, and the increased conversion of estrone to estradiol affects follicle development and raises the LH to FSH ratio, leading to ovulatory dysfunction [46]. Excessive androgens are produced by obese women’s extra adipose tissue; induce hirsutism, virilization, and hyperestrogenemia; and inhibit follicle-stimulating hormone [47]. Moreover, hypertrophic adipocytes are more prone to inflammation, apoptosis, fibrosis, and the release of free fatty acids [48]. PCOS is highly associated with a proinflammatory state since adipocytes are more sensitive to inflammatory cells, cytokines, and chemokines [49]. Androgens have been demonstrated to disrupt insulin signaling pathways, specifically in muscle and adipose tissues, resulting in decreased glucose uptake and hindered insulin function [50]. The excessive release of androgens is also linked to dysfunction in the islets of Langerhans, leading to impaired pancreatic metabolic processes that result in hyperinsulinemia, which is directly associated with an increased risk of type 2 diabetes mellitus (DM) [51]. Furthermore, hyperandrogenism worsens insulin resistance through various pathways, since it affects the expression of GLUT-4, and inhibits insulin degradation in the liver [51].

Leptin seems also to play a crucial role in regulating insulin resistance, due to its role in managing the energy balance and fat storage, and is secreted by adipocytes [52]. In women with PCOS, leptin levels are elevated due to an increased fat mass, but the body becomes resistant to its effects, similar to insulin resistance [53]. Several studies have reported that leptin levels are higher in overweight/obese women, indicating that this increase is because of the higher BMI in these cases [54]. Moreover, in a recent meta-analysis leptin levels are moderately elevated in non-obese PCOS women when compared to healthy BMI-matched controls, indicating that leptin levels are independently higher in women with PCOS [53]. Notably, another study reported an association between serum leptin levels and hyper-fasting serum insulin levels, demonstrating the correlation between leptin levels and insulin resistance in PCOS patients [55]. Elevated leptin levels contribute to resistance to leptin signaling and trafficking to leptin receptors, resulting in leptin resistance [56,57,58]. High levels shut down leptin signaling pathways, which accounts for obesity-induced infertility or subfertility in women with PCOS [56].

The pathogenesis of PCOS in lean patients exhibits unique features since it is mainly characterized by hormonal imbalances, ovulatory dysfunction, and metabolic disturbances [59]. Androgen overproduction and increased levels of LH overstimulate the ovaries, resulting in irregular menstruation, infertility, and secondary symptoms such as hirsutism, acne, and hair thinning [60]. Moreover, these patients show hypothalamic–pituitary–ovarian (HPO) axis dysregulation, which leads to the abnormal secretion of LH and follicle-stimulating hormone (FSH), disrupted menstrual cycles, and anovulation [61]. In some cases, increased adrenal androgen secretion acts synergistically with hyperandrogenism, due to altered adrenal steroidogenesis pathways [62]. In addition, lean patients with PCOS show disrupted folliculogenesis, since the ovarian follicles do not normally develop, and they demonstrate altered follicle sensitivity to gonadotropins (LH and FSH) [63].

**Figure 3:**
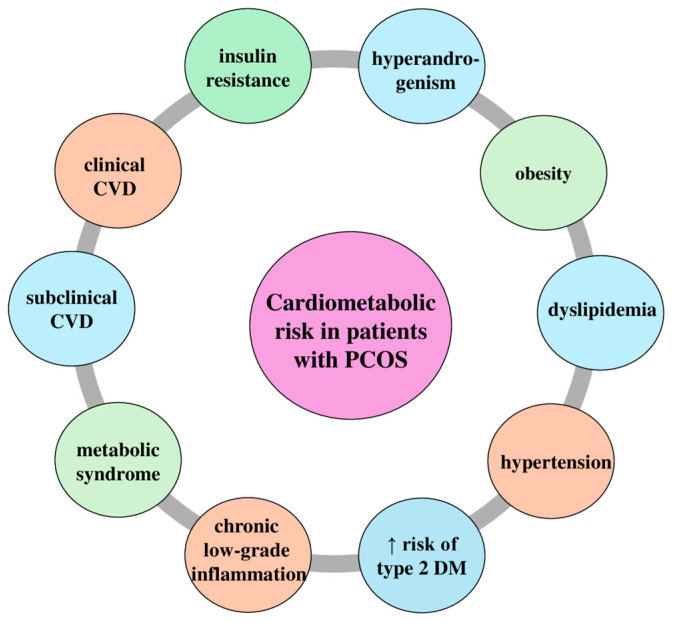
Cardiometabolic risk in patients with polycystic ovarian syndrome (PCOS). The key cardiometabolic risks associated with PCOS, including insulin resistance, hyperandrogenism, obesity, dyslipidemia, hypertension, increased risk of type 2 diabetes mellitus (DM), chronic low-grade inflammation, metabolic syndrome, subclinical cardiovascular disease (CVD), and clinical CVD. ↑: higher.

Finally, women with PCOS exhibit chronic low-grade inflammation that exacerbates insulin resistance [64]. The elevated levels of inflammatory cytokines, such as tumor necrosis factor-alpha (TNF-α) and interleukin-6 (IL-6), can impair the insulin signaling pathway by increasing the serine phosphorylation of IRS, and inducing the production of suppressors of cytokine signaling (SOCS) proteins, which disrupt insulin receptor function [65]. An additional impairment of insulin signaling pathways can also be induced by the higher levels of oxidative stress in women with PCOS [66]. These data reveal that insulin resistance in women with PCOS is multifactorial, and understanding the mechanism would be crucial for disease management (Figure 3).

### 2.2. Subclinical CVD and PCOS

Several studies have investigated the atherosclerotic alterations induced in women with PCOS [67,68,69,70]. These changes in blood vessels occur in the early stages of the disease, before any clinical symptoms develop, being a crucial therapeutic window to capture prevention before the disease sets in.

Endothelial dysfunction is an asymptomatic structural vascular alteration that is involved in the early subclinical stage of atherosclerotic diseases [71,72]. Flow-mediated dilation (FMD) is a non-invasive ultrasound technique used to assess endothelial function, helping in the early detection of cardiovascular risk and guiding therapeutic interventions aimed at improving vascular health [73,74,75]. A large study of 2264 asymptomatic post-menopausal women demonstrated that the lower FMD tertile was linked to a fourfold higher risk of adverse cardiovascular events [76]. A large meta-analysis of 21 studies with a total of 908 women with PCOS reported a 3.4% lower FMD in women of reproductive age with PCOS [77]. Moreover, a recent cross-sectional study that compared different groups of women with PCOS found that FMD in obese subjects was more impaired (9.2%) than in overweight subjects (13.7%), and even more than in normal weight (12.5%) PCOS women (33,112,268). This may also be related to hyperandrogenism in PCOS patients since elevated androgen levels have been linked to endothelial dysfunction in both postmenopausal women and transgender men [78]. Moreover, insulin has a vasodilatory effect that occurs secondary to the release of endothelium-derived nitric oxide (NO). As a result, endothelial dysfunction may worsen due to insulin resistance, which is associated with a reduction in the synthesis and release of NO [79].

**Figure 4:**
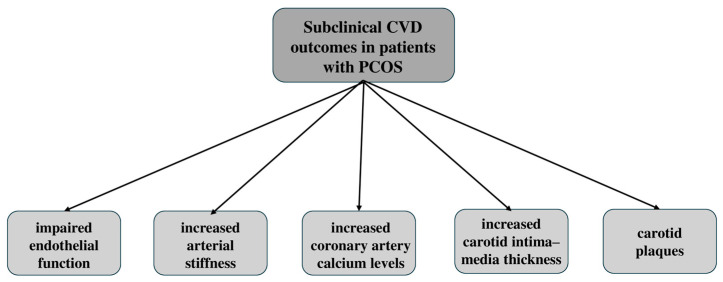
Subclinical cardiovascular disease (CVD) outcomes in patients with polycystic ovarian syndrome (PCOS). Subclinical CVD outcomes in women with PCOS, including impaired endothelial function, increased arterial stiffness, elevated coronary artery calcium levels, an increased carotid intima–media thickness, and the presence of carotid plaques.

Another subclinical atherosclerotic subclinical technique is the evaluation of coronary artery calcium (CAC). A large study of premenopausal women with PCOS compared to matched controls found that CAC was more common in women with PCOS with OR of 2.37 (0.99–5.73, *p* = 0.05) [80]. However, the data are mixed for CAC, as the large study of 2029 participants showed that CAC was not related to the free androgen index [81]. The free androgen index is highly associated with impaired arterial stiffness, as evaluated by pulse wave velocity (PWV) [82]. Moreover, obese adolescents with PCOS had a higher arterial stiffness compared to obese adolescent controls, indicating that early signs of cardiovascular disease may start as early as adolescence [83]. What is also worth noting is the increased epicardial fat thickness reported in women with PCOS, which is a marker of cardiometabolic risk that appears to be associated with hyperandrogenism in a recent case-control study [84]. Finally, a large meta-analysis demonstrated that women with PCOS were noted to have a higher carotid intima–media thickness, which is highly associated with an increased risk of CVD adverse events [85]. While research indicates a higher prevalence of subclinical CVD markers in women with PCOS, early management of the disease is crucial in preventing the progression of subclinical CVD to more severe conditions (Figure 4).

### 2.3. Clinical CVD and PCOS

Cardiovascular disease continues to be a major factor responsible for the vast majority of adverse events in women with PCOS, primarily affecting postmenopausal women, but being pivotal even in early adulthood [28]. However, the risk of CVD remains uncertain, since there is conflicting evidence about whether PCOS independently increases the risk of clinical CVD events.

According to a large meta-analysis of 166,682 subjects in total, the pooled risk of CVD events in PCOS women demonstrated a OR of 1.66 (95% CI: 1.32–2.08), and the risks of myocardial infarction (OR: 2.57, 95% CI: 1.37–4.82), ischemic heart disease (OR: 2.77, 95% CI: 2.12–3.61), and stroke (OR: 1.96, 95% CI: 1.56–2.47) were higher in the PCOS group, showing an overall increased risk of CVD events among PCOS patients [86]. Moreover, four cohort studies showed higher cardiovascular mortality among women with PCOS compared to healthy controls [20]. However, another meta-analysis of five case–control and five cohort studies reported that no significant association was observed between PCOS and myocardial infarction [26]. When compared to the midlife adverse CVD events in women with PCOS vs. healthy control in another large meta-analysis, the PCOS group had no higher risk of these events [31].

Large studies, such as a Taiwan National Health Insurance study with 8048 females aged 15–49 years with PCOS and 32,192 controls, reported a 63% greater risk of CVD events after a 5.9-year follow-up period [87]. Moreover, in terms of heart physiology, patients with PCOS exhibit a lower cardiac systolic flow velocity, and there is an inverse association between serum fasting insulin levels and left ventricular systolic outflow parameters when compared to healthy controls [88]. Additionally, a significant rise in the left ventricular mass index, which is a predictor of CVD-related morbidity and mortality, has also been observed in normal-weight patients with PCOS [89]. Finally, a large registry in Denmark found a greater CVD event rate for PCOS women (22.6 per 1000 patient-years) than healthy controls (13.2 per 1000 patient-years) [25]. These conflicting findings among studies may be caused by the heterogeneity of the population, the matching techniques, the age, and the BMI of women with PCOS. Further research is needed to fully determine the risk of cardiovascular disease among women with PCOS. However, a recent meta-analysis and update of the 2023 guidelines show a clear association, recognizing PCOS as a significant risk factor for CVD morbidity [90]. Particularly, according to this meta-analysis, which included 1.06 million women, women with PCOS have a 68% higher risk of developing any CVD, a 48% increased risk of ischemic heart disease, a 150% higher risk of myocardial infarction, and a 71% higher risk of stroke compared to women without PCOS [90] (Table 1).

## 3. Management of PCOS

### 3.1. Diet Intervention

A good nutritional status and a proper diet are crucial as therapeutic strategies for PCOS, as they are also important in preventing the disorder’s side effects [91]. Several studies have indicated that women with PCOS tend to consume more calories and saturated fats while having insufficient fiber intake [92]. These dietary alterations may exacerbate clinical symptoms and increase the compounded risk of chronic disease in patients with PCOS [93]. In women with PCOS and obesity, notable changes in the metabolism of carbohydrates, lipids, and amino acids have been observed, along with distinct metabolomic signatures [94]. These include reductions in citric acid, lactic acid, lysophosphatidylcholine, and glycerophosphocholine levels, as well as increases in the levels of free fatty acids like carnitine, adipic acid, linoleic acid, and oleic acid [95]. One of the primary objectives of medical nutrition therapy for women with PCOS is to reduce insulin resistance and enhance reproductive function [85]. Achieving a weight loss of 5 to 10% can significantly improve reproductive function, since it is believed that dietary nutrients can directly impact metabolic regulation, inflammation, and oxidative stress [96]. Various dietary patterns have been recommended for managing PCOS.

The Mediterranean diet (Med Diet) is considered one of the most effective non-pharmacological approaches for treating PCOS [97]. Research indicates that following the MedDiet can enhance ovarian health (including ovarian volume and follicle count) by addressing obesity, insulin resistance, and hyperandrogenism [98]. This is believed to be linked to the relationship between adherence to the MedDiet and the levels of sex hormone-binding globulin (SHBG) and endogenous estrogens in women [99]. The positive effects of the MedDiet are largely attributed to plant polyphenols that are found in vegetables, fruits, legumes, grains, nuts, seeds, and especially in red wine and extra-virgin olive oil and may help combat metabolic syndrome and have been extensively studied over the past several decades [98]. In women with PCOS, polyphenols may play roles in disease prevention and treatment by reducing inflammation, enhancing insulin sensitivity, and managing compensatory hyperinsulinemia [98]. The Mediterranean diet likely benefits women with PCOS by lowering inflammatory and oxidative stress markers and enhancing the lipid profile, insulin sensitivity, endothelial function, and anti-atherosclerotic and anti-thrombotic properties [100].

Another option is the ketogenic diet. Current data suggest that a very low-calorie ketogenic diet may be an effective short-term dietary intervention for treating PCOS [101]. The diet was designed to induce ketosis while maintaining lean mass with an adequate protein amount, without being classified as a high-protein diet [102]. The goal was to rapidly and significantly lower blood glucose and insulin levels while increasing glucagon levels to stimulate lipolysis, thereby promoting the liver’s production of ketones [103]. This approach leads to rapid weight loss and improvements in body composition and the metabolic profile, such as reductions in waist circumference, fat mass, blood glucose levels, and HbA1c levels, as well as enhanced insulin sensitivity, which are critical factors in the pathophysiology of PCOS [104]. The ketogenic diet regulates the menstrual cycle, reduces blood glucose levels and body weight, enhances liver function, and helps treat fatty liver [105]. One study found that within just six weeks, there was a significant improvement in insulin resistance, a reduction in fat mass, and decreased acyclic estrogen production from the aromatization of excess androgens in adipose tissue, leading to a better LH/FSH ratio [106]. In a recent retrospective study that evaluated the impact of a very-low-calorie ketogenic diet on markers predictive of metabolic and ovulatory dysfunction in women with obesity and PCOS over 12 weeks, a significant decrease in serum anti-Müllerian hormone levels and significant increases in progesterone and sex hormone-binding globulin levels were observed [104].

Another lifestyle change is the low-glycemic index (GI) diet, which primarily derives carbohydrates from low-glycemic index sources, which has gained popularity among both registered dietitians and patients for managing PCOS. A low-GI diet may help reduce inflammation in PCOS patients by increasing uric acid levels and enhancing glutathione peroxidase activity, whose levels tend to be lower in women with PCOS [98]. Low-glycemic index (low-GI) diets may affect hormones that regulate appetite, such as ghrelin and glucagon, since these meals produced decreased ghrelin levels and increased glucagon levels in women with PCOS [107]. Recent studies highlight low-GI diets as an optimal dietary option for women with PCOS due to their high adherence rates and effectiveness in addressing common PCOS symptoms, including insulin resistance, hyperandrogenism, hirsutism, acne, and menstrual irregularities [101]. Furthermore, one study found that low-GI diets positively affect anthropometric and metabolic characteristics in both overweight women with and without PCOS [98]. The significance of a dietary approach is now evident. While drug therapy has shown short-term effectiveness, a combination of a tailored diet and a regular exercise routine is likely the only method that offers sustainable results.

### 3.2. Metformin

The link between PCOS and the components of metabolic syndrome, such as central obesity, dyslipidemia, hypertension, and glucose intolerance, explains the established connection with type 2 DM, CVD, and hormone-responsive cancers later in life [108]. High androgen levels in patients with PCOS are highly affected by hyperinsulinemia and insulin resistance since insulin regulates ovarian function, and excessive insulin can negatively impact the ovaries [8]. Medications like metformin, an insulin sensitizer, have been shown to promote ovulation by lowering insulin resistance, reducing circulating androgen levels, and improving menstrual regularity [105]. Metformin is an oral antidiabetic medication with insulin-sensitizing properties. Insulin resistance is reported in over 75% of lean women and 95% of overweight women with PCOS [109]. It is also believed to have direct anti-inflammatory effects by inhibiting nuclear factor κB (NF-κB) activation and reducing the release of inflammatory cytokines like interleukin-6 (IL-6), as well as indirectly by lowering hyperglycemia and insulin levels [110]. In non-pregnant women with PCOS, metformin enhances ovulation, improves pregnancy rates, reduces insulin resistance, and lowers androgen levels [111].

Metformin has several potential effects, including enhancing glucose uptake, improving insulin signaling, reducing fatty acid and triglyceride synthesis, and increasing fatty acid β-oxidation. It may also boost glucose utilization in peripheral tissues and possibly reduce food intake and intestinal glucose absorption [112]. Unlike other antidiabetic drugs, metformin does not promote the secretion of endogenous insulin, and so it does not cause hypoglycemia or hyperinsulinemia [113]. By enhancing insulin sensitivity, metformin lowers insulin levels and subsequently decreases circulating androgen levels [113]. Metformin also improves insulin-mediated glucose disposal in women with PCOS, making it a key medication in treating the condition [114]. Moreover, metformin could also alter the metabolic capacity of ovarian granulosa cells by indirectly regulating developing oocytes, since it reduces androgen levels in the bloodstream and follicular environment of women with PCOS [115]. Furthermore, a large meta-analysis showed that oral metformin administration resulted in a mean reduction in BMI of approximately 0.73 kg/m^2^ after six months and a clinically significant reduction in waist circumference [116]. Metformin enhances insulin sensitivity and, as previously mentioned, has been proven to slow or prevent the progression to type 2 DM by 26% in individuals with impaired glucose tolerance [117]. While it has not been specifically shown to lower the risk of cardiovascular events in patients with PCOS, existing mechanistic and clinical evidence supports its use as a protective measure against the cardiovascular risks associated with insulin resistance and excess insulin [21]. Finally, according to the recent 2023 guidelines, the evidence indicates that metformin is recommended for specific groups, especially those women with clinical metabolic symptoms, due to its efficiency in improving weight, BMI, the waist–hip ratio, testosterone levels, and triglyceride levels in women with PCOS [14].

### 3.3. Glucagon-like Peptide-1 Receptor Agonists (GLP-1RAs)

Obesity is a major metabolic concern for individuals with PCOS, affecting an estimated 50% or more of this population [118,119]. Additionally, insulin resistance is a key feature of PCOS, impacting about 50–80% of those with this disorder [59]. Weight loss has been shown to improve hyperandrogenism, reproductive function, and metabolic parameters such as hyperlipidemia, glycemic control, and hypertension in women with PCOS [120]. Weight loss has mixed effects on fertility in women with PCOS, since in a recent study of obese infertile women, an average 6-month loss of 10 kg resulted in the restoration of ovulatory function in 90% of them, of whom 78% became pregnant, with a miscarriage rate of 18% [121]. Moreover, in a large randomized trial of infertile women with PCOS, the intervention group (5% weight loss in 24 months) was compared with the control group (no weight loss) and reported 27.1% and 35.2% vaginal births, respectively [122]. Despite these mixed outcomes, weight loss before conception in women with obesity and PCOS can reduce pregnancy-related risks [123].

Glucagon-like peptide-1 receptor agonists (GLP-1RAs) have recently become a valuable option for the metabolic management of polycystic ovarian syndrome (PCOS) [124]. GLP-1RAs are a group of medications originally developed for treating type 2 diabetes mellitus, which enhance insulin secretion in a glucose-dependent manner [125]. The primary action of GLP-1RAs mirrors that of natural GLP-1, as all synthetic GLP-1RAs bind to the GLP-1 receptor, triggering insulin release from pancreatic islets in response to glucose [125]. GLP-1 analogs also offer additional benefits, such as slowing gastric emptying and suppressing glucagon production by pancreatic alpha cells [126]. GLP-1RAs have been effective in lowering glycated hemoglobin levels, supporting weight loss, and improving hyperlipidemia [127]. Also, GLP-1RAs play a catalytic role in improving endothelial function by enhancing nitric oxide (NO) production, reducing blood pressure, lowering triglyceride and LDL cholesterol levels, and ameliorating left ventricular function, contributing to an overall cardiovascular benefit [125,128,129,130].

Several studies have investigated the role of GLP-RAs in women with PCOS. Particularly, a trial involving 72 overweight women with PCOS treated with liraglutide at a dose of 1.8 mg/day or a placebo for 26 weeks demonstrated that liraglutide significantly reduced body weight by more than 5%, liver fat by 44%, visceral fat by 18%, and free testosterone levels by 19% [131]. A clinical study closely monitored ovarian changes using ultrasonography in obese PCOS patients treated with GLP-1RAs for 6 months, revealing a significant reduction in ovarian volume compared to those in the placebo group [132]. Furthermore, a large meta-analysis of eight randomized control trials reported that GLP-1RAs were more effective in improving insulin sensitivity and body mass index than metformin [133]. However, combined treatment with GLP-1RAs and metformin demonstrated significant advantages in weight loss, waist circumference, fasting blood glucose levels, and fasting insulin levels, while the incidence of adverse CVD reactions was relatively high [134]. Additionally, a meta-analysis of 11 RCTs revealed that the usage of GLP-1RAs is associated with improvements in the natural pregnancy rate, menstrual regularity, insulin sensitivity, anthropometrics, and hormonal indexes in PCOS women [135]. Finally, other benefits included enhanced psychological well-being, higher remission rates of prediabetes, reductions in free testosterone levels and the free androgen index, improvements in dyslipidemia, and reductions in ectopic fat accumulation, which led to better glucose regulation, reduced inflammation, and improved cardiovascular health [136].

### 3.4. Oral Contraceptives

Hormonal contraceptives, such as oral contraceptives, the patch, or the vaginal ring, are the primary treatment approaches for managing menstrual irregularities and hirsutism or acne in women with PCOS [137]. Exogenous estrogen provides feedback control of follicle-stimulating hormone (FSH), preventing ovulation and thickening the uterine lining [138]. Combined oral contraceptive pills (COCPs) are most commonly available as combined formulations of estrogen and progestin [139]. Moreover, oral contraceptives provide direct negative feedback on LH secretion, leading to reduced ovarian production of androgens and consequently lowering hyperandrogenism and raising sex hormone-binding globulin levels in the liver, which decreases the amount of free circulating androgens [140]. Additional mechanisms of action for oral contraceptives include inhibiting the peripheral conversion of testosterone to dihydrotestosterone, preventing dihydrotestosterone from binding to androgen receptors, and decreasing adrenal androgen secretion [141]. However, metformin was inferior in the free androgen index, insulin secretion, plasma triglyceride levels, sex hormone-binding globulin (SHBG) levels, and testosterone levels compared with COCPs according to a meta-analysis of 36 RCTs [142]. Furthermore, the combination of COCPs with metformin significantly reduced fasting glucose and fasting insulin levels compared with COCPs alone [143]. Finally, their effects on women’s metabolic profiles are mixed since there are absolute and relative contraindications and risks and benefits in the general population. Their benefits are greater when combined with metformin therapy according to 2023 International Guidelines [14].

### 3.5. Myo-Inositol (MI)

Inositols are part of the vitamin B complex produced naturally within the human body [144]. There are nine stereoisomers, with myoinositol (MI) and D-chiro-inositol being the most important [145]. Inositols are regarded as insulin sensitizers because they regulate components of insulin signaling pathways [146]. Several studies have demonstrated that one mechanism of insulin deficiency involves the inositolphosphoglycan (IPG) mediator, and a lack of inositol in IPGs is linked to insulin resistance [147]. They have beneficial effects on menstrual cycle regularity, carbohydrate metabolism, and the clinical and laboratory signs of hyperandrogenism (such as free testosterone, total testosterone, and SHBG levels) [148]. Particularly, a recent meta-analysis of nine RCTs involving 247 PCOS subjects and 249 controls demonstrated that 12–24 weeks of MI treatment resulted in significant decreases in fasting insulin levels and the homeostasis model assessment (HOMA) index, and a trend toward a reduced testosterone concentration [149]. Moreover, the administration of MI significantly increased SHBG levels after at least 24 weeks [149]. Another meta-analysis including ten RCTs (573 patients with PCOS) confirmed the results of improving HOMA index and raising estradiol levels [150]. Also, a recent RCT study revealed that when comparing 1200 mg of MI vs. placebo for 12 weeks in women with a BMI < 25 kg/m^2^, no significant reductions were reported in BMI, testosterone levels, and fasting insulin levels, showing that MI’s cardiometabolic effects are conflicting [151]. Additionally, when comparing metformin vs. MI, the results obtained from both groups were similar since the insulin sensitivity was improved in both groups, BMI significantly decreased, and the menstrual cycle was normalized in approximately 50% of the women [152]. A recent meta-analysis included eight studies with a total sample size of 1088 and compared metformin and MI, which also indicated that there were no significant differences in BMI, fasting insulin levels, fasting blood sugar levels, the HOMA index, and the LH/FSH ratio [153]. The recent 2023 guidelines characterize the benefit of inositol as inadequate to make an evidence-based recommendation on efficacy for clinical outcomes, but it has “potential” for improvements in cardiometabolic metabolic measures and limited effectiveness for ovulation and hirsutism [14].

### 3.6. Bariatric Surgery

**Figure 5:**
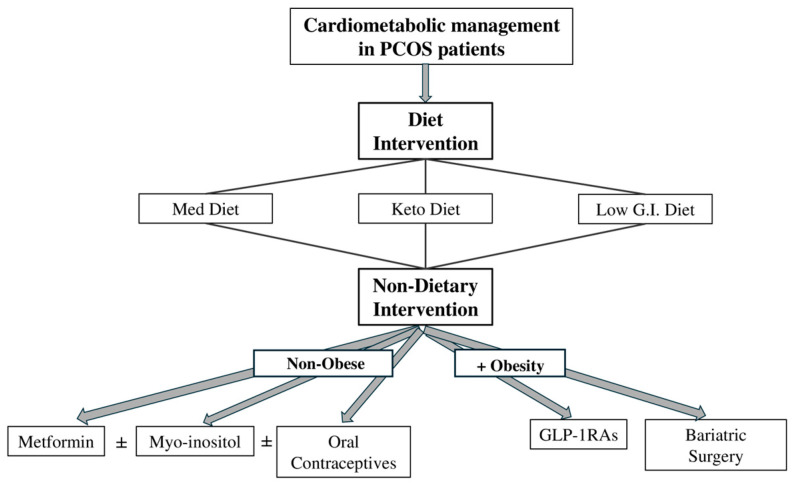
Cardiometabolic management in patients with polycystic ovarian syndrome (PCOS). Primary individualized interventions focusing on dietary changes are administered as the [Mediterranean diet (Med Diet), ketogenic diet (Keto Diet) or low-glycemic index (Low G.I.) diet] and secondary non-dietary interventions, depending on the body mass index. The non-dietary interventions are distinguished between obese and non-obese individuals: (i) non-obese—metformin and/or myo-inositol and/or oral contraceptives; and (ii) obese—glucagon-like peptide-1 receptor agonists (GLP-1RAs) and/or bariatric surgery. ±: combined treatment or separate treatment, individually per patient medical history.

The management of PCOS primarily addresses the symptoms rather than the underlying causes. Oral contraceptives are commonly used because they help alleviate symptoms such as excess androgen levels and irregular bleeding, and prevent the thickening of the uterine lining [154]. However, in women with obesity, PCOS can often be improved or even reversed through weight loss. In women with a BMI > 35 kg/m^2^, lifestyle changes often fail to produce long-term results, while bariatric or metabolic surgery has been proven to offer significant and lasting weight loss, along with improvements in overall metabolic health and quality of life. The most frequently performed surgeries, involving minimally invasive techniques, are Vertical Sleeve Gastrectomy and Roux-en-Y gastric bypass [155]. Although these surgical techniques have persistent benefits and superior weight loss, they can lead to nutrient deficiencies that are crucial for fetal development and are associated with higher risks of perinatal mortality and preterm births [156]. The substantial weight loss following bariatric surgery has been shown to reduce insulin resistance, which may partly account for the improvement in menstrual regularity observed after surgery [156]. Additionally, weight loss may help reverse hyperandrogenism in patients with PCOS by increasing SHBG levels, thereby lowering circulating androgen levels [156]. Several studies have indicated that while lifestyle changes or medication may lead to significant improvements in other aspects of PCOS, ovarian morphology often shows either no substantial improvement or only minor changes [157]. A large meta-analysis of 13 studies with 2130 female patients indicated that the incidence of PCOS preoperatively was 45.6% and postoperatively was 6.8% at 12 months after bariatric surgery [158]. Moreover, menstrual irregularity and hirsutism both significantly decreased at 12 months [158]. Another meta-analysis of nine studies with 234 obese PCOS patients reported that bariatric surgery reduced the total testosterone level by 25.82 mg/dl, serum free testosterone level by 4.10 ng/dL, and BMI by 14.51 kg/m^2^ in 12-month follow-up, showing also significant effect on the type 2 DM and hypertension relative risks [159]. Finally, according to a large prospective observational study of 1013 PCOS patients, surgery leads to significant weight loss and resolves many PCOS-related issues, such as hirsutism and menstrual irregularities, since women experienced a complete resolution of these symptoms within 6 months to 2 years post-surgery, and it is an effective treatment option for enhancing metabolic health and improving menstrual regularity and hirsutism [160] (Figure 5).

## 4. Conclusions

In conclusion, PCOS is a diverse syndrome with several variations in clinical symptoms and pathogenetic mechanisms among different phenotypes, contributing to its complexity, particularly in the diagnosis and treatment of its long-term risks, such as cardiovascular disease and insulin resistance. Although treatments like metformin, GLP-1RAs, dietary modifications, hormonal contraceptives, and bariatric surgery show efficacy in managing metabolic and reproductive symptoms, the long-term management of PCOS requires individualized care. Identifying PCOS early and addressing its cardiometabolic risks is crucial for improving the patient’s quality of life.

## Figures and Tables

**Table 1:** Included studies investigating the clinical cardiovascular risk in patients with polycystic ovarian syndrome (PCOS).

Study	Key Findings in PCOS Subjects
Zhang et al., 2020 [86]	↑ Risk of CVD events (OR: 1.66) ↑ Risk of myocardial infarction (OR: 2.57) ↑ Risk of ischemic heart disease (OR: 2.77) ↑ Risk of stroke (OR: 1.96)
Wekker et al., 2020 [20]	↑ Risk of CVD mortality (RR: 1.78)
Zhao et al., 2016 [26]	~ No risk of myocardial Infarction (OR = 1.01)
Iftikhar et al., 2012 [31]	~ No risk of CVD events (*p* = 0.16)
Ding et al., 2018 [87]	↑ 63% greater risk of CVD
Prelevic et al., 1995 [88]	↓ Cardiac systolic flow velocity
Yarali et al., 2001 [89]	↑ Left ventricular mass index ↑ Cardiac diastolic dysfunction
Glintborg et al., 2018 [25]	22.6 CVD events per 1000 patient-years in patients with PCOS vs. 13.2 per 1000 patient-years in controls

PCOS: polycystic ovarian syndrome, CVD: cardiovascular disease, OR: odds ratio, RR: relative risk, ↑: increased risk, ↓: reduced risk, ~: neither higher not lower risk.

## Data Availability

Not applicable.

## References

[1] Andrews M.C. (1952). Bilateral polycystic ovaries associated with sterility, amenorrhea and hirsutism. Va. Med. Mon..

[2] Diamanti-Kandarakis E., Kouli C.R., Bergiele A.T., Filandra F.A., Tsianateli T.C., Spina G.G., Zapanti E.D., Bartzis M.I. (1999). A survey of the polycystic ovary syndrome in the Greek island of Lesbos: Hormonal and metabolic profile. J. Clin. Endocrinol. Metab..

[3] Azziz R., Woods K.S., Reyna R., Key T.J., Knochenhauer E.S., Yildiz B.O. (2004). The prevalence and features of the polycystic ovary syndrome in an unselected population. J. Clin. Endocrinol. Metab..

[4] Yildiz B.O., Bozdag G., Yapici Z., Esinler I., Yarali H. (2012). Prevalence, phenotype and cardiometabolic risk of polycystic ovary syndrome under different diagnostic criteria. Hum. Reprod..

[5] Sirmans S.M., Pate K.A. (2013). Epidemiology, diagnosis, and management of polycystic ovary syndrome. Clin. Epidemiol..

[6] Agapova S.E., Cameo T., Sopher A.B., Oberfield S.E. (2014). Diagnosis and challenges of polycystic ovary syndrome in adolescence. Semin. Reprod. Med..

[7] Dewailly D., Lujan M.E., Carmina E., Cedars M.I., Laven J., Norman R.J., Escobar-Morreale H.F. (2014). Definition and significance of polycystic ovarian morphology: A task force report from the Androgen Excess and Polycystic Ovary Syndrome Society. Hum. Reprod. Update.

[8] Rosenfield R.L., Ehrmann D.A. (2016). The Pathogenesis of Polycystic Ovary Syndrome (PCOS): The Hypothesis of PCOS as Functional Ovarian Hyperandrogenism Revisited. Endocr. Rev..

[9] Lobo R.A. (1995). A disorder without identity: “HCA,” “PCO,” “PCOD,” “PCOS,” “SLS”. what are we to call it?!. Fertil. Steril..

[10] Behera M., Price T., Walmer D. (2006). Estrogenic ovulatory dysfunction or functional female hyperandrogenism: An argument to discard the term polycystic ovary syndrome. Fertil. Steril..

[11] Sam S., Dunaif A. (2003). Polycystic ovary syndrome: Syndrome XX?. Trends Endocrinol. Metab..

[12] Legro R.S., Arslanian S.A., Ehrmann D.A., Hoeger K.M., Murad M.H., Pasquali R., Welt C.K., Endocrine S. (2013). Diagnosis and treatment of polycystic ovary syndrome: An Endocrine Society clinical practice guideline. J. Clin. Endocrinol. Metab..

[13] The Rotterdam ESHRE/ASRM-Sponsored PcOS Consensus workshop Group (2004). Revised 2003 consensus on diagnostic criteria and long-term health risks related to Polycystic ovary syndrome (PCOS). Hum. Reprod..

[14] Teede H.J., Tay C.T., Laven J.J.E., Dokras A., Moran L.J., Piltonen T.T., Costello M.F., Boivin J., Redman L.M., Boyle J.A. (2023). Recommendations From the 2023 International Evidence-based Guideline for the Assessment and Management of Polycystic Ovary Syndrome. J Clin. Endocrinol. Metab..

[15] Lizneva D., Suturina L., Walker W., Brakta S., Gavrilova-Jordan L., Azziz R. (2016). Criteria, prevalence, and phenotypes of polycystic ovary syndrome. Fertil. Steril..

[16] Baldani D.P., Skrgatic L., Simunic V., Zlopasa G., Canic T., Trgovcic I. (2013). Characteristics of different phenotypes of polycystic ovary syndrome based on the Rotterdam criteria in the Croatian population. Coll. Antropol..

[17] Mehrabian F., Khani B., Kelishadi R., Kermani N. (2011). The prevalence of metabolic syndrome and insulin resistance according to the phenotypic subgroups of polycystic ovary syndrome in a representative sample of Iranian females. J. Res. Med. Sci..

[18] Hsu M.I., Liou T.H., Chou S.Y., Chang C.Y., Hsu C.S. (2007). Diagnostic criteria for polycystic ovary syndrome in Taiwanese Chinese women: Comparison between Rotterdam 2003 and NIH 1990. Fertil Steril.

[19] Bozdag G., Mumusoglu S., Zengin D., Karabulut E., Yildiz B.O. (2016). The prevalence and phenotypic features of polycystic ovary syndrome: A systematic review and meta-analysis. Hum. Reprod..

[20] Wekker V., van Dammen L., Koning A., Heida K.Y., Painter R.C., Limpens J., Laven J.S.E., Roeters van Lennep J.E., Roseboom T.J., Hoek A. (2020). Long-term cardiometabolic disease risk in women with PCOS: A systematic review and meta-analysis. Hum. Reprod. Update.

[21] Guan C., Zahid S., Minhas A.S., Ouyang P., Vaught A., Baker V.L., Michos E.D. (2022). Polycystic ovary syndrome: A “risk-enhancing” factor for cardiovascular disease. Fertil. Steril..

[22] Rizzo M., Berneis K., Hersberger M., Pepe I., Di Fede G., Rini G.B., Spinas G.A., Carmina E. (2009). Milder forms of atherogenic dyslipidemia in ovulatory versus anovulatory polycystic ovary syndrome phenotype. Hum. Reprod..

[23] Hart R., Doherty D.A. (2015). The potential implications of a PCOS diagnosis on a woman’s long-term health using data linkage. J. Clin. Endocrinol. Metab..

[24] de Groot P.C., Dekkers O.M., Romijn J.A., Dieben S.W., Helmerhorst F.M. (2011). PCOS, coronary heart disease, stroke and the influence of obesity: A systematic review and meta-analysis. Hum. Reprod. Update.

[25] Glintborg D., Rubin K.H., Nybo M., Abrahamsen B., Andersen M. (2018). Cardiovascular disease in a nationwide population of Danish women with polycystic ovary syndrome. Cardiovasc. Diabetol..

[26] Zhao L., Zhu Z., Lou H., Zhu G., Huang W., Zhang S., Liu F. (2016). Polycystic ovary syndrome (PCOS) and the risk of coronary heart disease (CHD): A meta-analysis. Oncotarget.

[27] Gui J., Wang R.H. (2017). Cardiovascular autonomic dysfunction in women with polycystic ovary syndrome: A systematic review and meta-analysis. Reprod. Biomed. Online.

[28] Ramezani Tehrani F., Amiri M., Behboudi-Gandevani S., Bidhendi-Yarandi R., Carmina E. (2020). Cardiovascular events among reproductive and menopausal age women with polycystic ovary syndrome: A systematic review and meta-analysis. Gynecol. Endocrinol..

[29] Toulis K.A., Goulis D.G., Mintziori G., Kintiraki E., Eukarpidis E., Mouratoglou S.A., Pavlaki A., Stergianos S., Poulasouchidou M., Tzellos T.G. (2011). Meta-analysis of cardiovascular disease risk markers in women with polycystic ovary syndrome. Hum. Reprod. Update.

[30] Zhou Y., Wang X., Jiang Y., Ma H., Chen L., Lai C., Peng C., He C., Sun C. (2017). Association between polycystic ovary syndrome and the risk of stroke and all-cause mortality: Insights from a meta-analysis. Gynecol. Endocrinol..

[31] Iftikhar S., Collazo-Clavell M.L., Roger V.L., St Sauver J., Brown R.D., Cha S., Rhodes D.J. (2012). Risk of cardiovascular events in patients with polycystic ovary syndrome. Neth. J. Med..

[32] Dapas M., Lin F.T.J., Nadkarni G.N., Sisk R., Legro R.S., Urbanek M., Hayes M.G., Dunaif A. (2020). Distinct subtypes of polycystic ovary syndrome with novel genetic associations: An unsupervised, phenotypic clustering analysis. PLoS Med..

[33] Cassar S., Misso M.L., Hopkins W.G., Shaw C.S., Teede H.J., Stepto N.K. (2016). Insulin resistance in polycystic ovary syndrome: A systematic review and meta-analysis of euglycaemic-hyperinsulinaemic clamp studies. Hum. Reprod..

[34] Tosi F., Bonora E., Moghetti P. (2017). Insulin resistance in a large cohort of women with polycystic ovary syndrome: A comparison between euglycaemic-hyperinsulinaemic clamp and surrogate indexes. Hum. Reprod..

[35] Zhu S., Zhang B., Jiang X., Li Z., Zhao S., Cui L., Chen Z.J. (2019). Metabolic disturbances in non-obese women with polycystic ovary syndrome: A systematic review and meta-analysis. Fertil. Steril..

[36] Liu Y.N., Qin Y., Wu B., Peng H., Li M., Luo H., Liu L.L. (2022). DNA methylation in polycystic ovary syndrome: Emerging evidence and challenges. Reprod. Toxicol..

[37] Sulaiman M.A., Al-Farsi Y.M., Al-Khaduri M.M., Saleh J., Waly M.I. (2018). Polycystic ovarian syndrome is linked to increased oxidative stress in Omani women. Int. J. Womens Health.

[38] Armanini D., Boscaro M., Bordin L., Sabbadin C. (2022). Controversies in the Pathogenesis, Diagnosis and Treatment of PCOS: Focus on Insulin Resistance, Inflammation, and Hyperandrogenism. Int. J. Mol. Sci..

[39] Sadeghi H.M., Adeli I., Calina D., Docea A.O., Mousavi T., Daniali M., Nikfar S., Tsatsakis A., Abdollahi M. (2022). Polycystic Ovary Syndrome: A Comprehensive Review of Pathogenesis, Management, and Drug Repurposing. Int. J. Mol. Sci..

[40] Oikonomou E., Tsaplaris P., Anastasiou A., Xenou M., Lampsas S., Siasos G., Pantelidis P., Theofilis P., Tsatsaragkou A., Katsarou O. (2022). Interleukin-1 in Coronary Artery Disease. Curr. Top. Med. Chem..

[41] Niswender K.D. (2011). Basal insulin: Physiology, pharmacology, and clinical implications. Postgrad. Med..

[42] Pierre A., Taieb J., Giton F., Grynberg M., Touleimat S., El Hachem H., Fanchin R., Monniaux D., Cohen-Tannoudji J., di Clemente N. (2017). Dysregulation of the Anti-Mullerian Hormone System by Steroids in Women with Polycystic Ovary Syndrome. J. Clin. Endocrinol. Metab..

[43] Salilew-Wondim D., Wang Q., Tesfaye D., Schellander K., Hoelker M., Hossain M.M., Tsang B.K. (2015). Polycystic ovarian syndrome is accompanied by repression of gene signatures associated with biosynthesis and metabolism of steroids, cholesterol and lipids. J. Ovarian Res..

[44] Ibanez L., Oberfield S.E., Witchel S., Auchus R.J., Chang R.J., Codner E., Dabadghao P., Darendeliler F., Elbarbary N.S., Gambineri A. (2017). An International Consortium Update: Pathophysiology, Diagnosis, and Treatment of Polycystic Ovarian Syndrome in Adolescence. Horm. Res. Paediatr..

[45] Lim J.J., Han C.Y., Lee D.R., Tsang B.K. (2017). Ring Finger Protein 6 Mediates Androgen-Induced Granulosa Cell Proliferation and Follicle Growth via Modulation of Androgen Receptor Signaling. Endocrinology.

[46] Li Y., Chen C., Ma Y., Xiao J., Luo G., Li Y., Wu D. (2019). Multi-system reproductive metabolic disorder: Significance for the pathogenesis and therapy of polycystic ovary syndrome (PCOS). Life Sci..

[47] Spritzer P.M., Lecke S.B., Satler F., Morsch D.M. (2015). Adipose tissue dysfunction, adipokines, and low-grade chronic inflammation in polycystic ovary syndrome. Reproduction.

[48] Villa J., Pratley R.E. (2011). Adipose tissue dysfunction in polycystic ovary syndrome. Curr. Diab Rep..

[49] Abraham Gnanadass S., Divakar Prabhu Y., Valsala Gopalakrishnan A. (2021). Association of metabolic and inflammatory markers with polycystic ovarian syndrome (PCOS): An update. Arch. Gynecol. Obstet..

[50] Yang F., Ruan Y.C., Yang Y.J., Wang K., Liang S.S., Han Y.B., Teng X.M., Yang J.Z. (2015). Follicular hyperandrogenism downregulates aromatase in luteinized granulosa cells in polycystic ovary syndrome women. Reproduction.

[51] Rodrigues J.K., Navarro P.A., Zelinski M.B., Stouffer R.L., Xu J. (2015). Direct actions of androgens on the survival, growth and secretion of steroids and anti-Mullerian hormone by individual macaque follicles during three-dimensional culture. Hum. Reprod..

[52] Obradovic M., Sudar-Milovanovic E., Soskic S., Essack M., Arya S., Stewart A.J., Gojobori T., Isenovic E.R. (2021). Leptin and Obesity: Role and Clinical Implication. Front. Endocrinol.

[53] Zheng S.H., Du D.F., Li X.L. (2017). Leptin Levels in Women with Polycystic Ovary Syndrome: A Systematic Review and a Meta-Analysis. Reprod. Sci..

[54] Pehlivanov B., Mitkov M. (2009). Serum leptin levels correlate with clinical and biochemical indices of insulin resistance in women with polycystic ovary syndrome. Eur. J. Contracept. Reprod. Health Care.

[55] Peng Y., Yang H., Song J., Feng D., Na Z., Jiang H., Meng Y., Shi B., Li D. (2022). Elevated Serum Leptin Levels as a Predictive Marker for Polycystic Ovary Syndrome. Front Endocrinol.

[56] Wolodko K., Walewska E., Adamowski M., Castillo-Fernandez J., Kelsey G., Galvao A. (2020). Leptin Resistance in the Ovary of Obese Mice is Associated with Profound Changes in the Transcriptome of Cumulus Cells. Cell Physiol. Biochem..

[57] Peelman F., Zabeau L., Moharana K., Savvides S.N., Tavernier J. (2014). 20 years of leptin: Insights into signaling assemblies of the leptin receptor. J. Endocrinol..

[58] Childs G.V., Odle A.K., MacNicol M.C., MacNicol A.M. (2021). The Importance of Leptin to Reproduction. Endocrinology.

[59] Diamanti-Kandarakis E., Dunaif A. (2012). Insulin resistance and the polycystic ovary syndrome revisited: An update on mechanisms and implications. Endocr. Rev..

[60] Fighera T.M., Dos Santos B.R., Spritzer P.M. (2023). Lean mass and associated factors in women with PCOS with different phenotypes. PLoS ONE.

[61] Doi S.A., Towers P.A., Scott C.J., Al-Shoumer K.A. (2005). PCOS: An ovarian disorder that leads to dysregulation in the hypothalamic-pituitary-adrenal axis?. Eur. J. Obstet. Gynecol. Reprod. Biol..

[62] Goodman N.F., Cobin R.H., Futterweit W., Glueck J.S., Legro R.S., Carmina E. (2015). American Association of Clinical Endocrinologists, American College of Endocrinology, and Androgen Excess and PCOS Society Disease State Clinical Review: Guide to the Best Practices in the Evaluation and Treatment of Polycystic Ovary Syndrome—Part 1. Endocr. Pr..

[63] Naamneh Elzenaty R., du Toit T., Fluck C.E. (2022). Basics of androgen synthesis and action. Best. Pract. Res. Clin. Endocrinol. Metab..

[64] Kwon H., Pessin J.E. (2013). Adipokines mediate inflammation and insulin resistance. Front Endocrinol.

[65] Xiang Y., Wang H., Ding H., Xu T., Liu X., Huang Z., Wu H., Ge H. (2023). Hyperandrogenism drives ovarian inflammation and pyroptosis: A possible pathogenesis of PCOS follicular dysplasia. Int. Immunopharmacol..

[66] Dabravolski S.A., Nikiforov N.G., Eid A.H., Nedosugova L.V., Starodubova A.V., Popkova T.V., Bezsonov E.E., Orekhov A.N. (2021). Mitochondrial Dysfunction and Chronic Inflammation in Polycystic Ovary Syndrome. Int. J. Mol. Sci..

[67] Yalcin Bahat P., Ozel A., Demirci A. (2021). Evaluation of Carotid Artery Intima-Media Thickness as a Cardiovascular Risk Factor in Patients with Polycystic Ovary Syndrome. Cureus.

[68] Krentowska A., Lebkowska A., Jacewicz-Swiecka M., Hryniewicka J., Lesniewska M., Adamska A., Kowalska I. (2021). Metabolic syndrome and the risk of cardiovascular complications in young patients with different phenotypes of polycystic ovary syndrome. Endocrine.

[69] Pandurevic S., Bergamaschi L., Pizzi C., Patton L., Rucci P., Corzani F., Cecchetti C., Pelusi C., Altieri P., Vicennati V. (2021). Body mass index rather than the phenotype impacts precocious ultrasound cardiovascular risk markers in polycystic ovary syndrome. Eur. J. Endocrinol..

[70] Jabbour R., Ott J., Eppel W., Frigo P. (2020). Carotid intima-media thickness in polycystic ovary syndrome and its association with hormone and lipid profiles. PLoS ONE.

[71] Oikonomou E., Siasos G., Tsigkou V., Bletsa E., Panoilia M.E., Oikonomou I.N., Sinanidis I., Spinou M., Papastavrou A., Kokosias G. (2020). Coronary Artery Disease and Endothelial Dysfunction: Novel Diagnostic and Therapeutic Approaches. Curr. Med. Chem..

[72] Thijssen D.H.J., Bruno R.M., van Mil A., Holder S.M., Faita F., Greyling A., Zock P.L., Taddei S., Deanfield J.E., Luscher T. (2019). Expert consensus and evidence-based recommendations for the assessment of flow-mediated dilation in humans. Eur. Heart J..

[73] Siasos G., Zografos T., Oikonomou E., Papavassiliou A.G., Stefanadis C., Tousoulis D. (2015). Flow-mediated dilation: Is it just a research tool or a useful biomarker for cardiovascular prognosis. Int. J. Cardiol..

[74] Oikonomou E., Souvaliotis N., Lampsas S., Siasos G., Poulakou G., Theofilis P., Papaioannou T.G., Haidich A.B., Tsaousi G., Ntousopoulos V. (2022). Endothelial dysfunction in acute and long standing COVID-19: A prospective cohort study. Vascul Pharmacol..

[75] Oikonomou E., Theofilis P., Lampsas S., Katsarou O., Kalogeras K., Marinos G., Tsatsaragkou A., Anastasiou A., Lysandrou A., Gounaridi M.I. (2022). Current Concepts and Future Applications of Non-Invasive Functional and Anatomical Evaluation of Coronary Artery Disease. Life.

[76] Rossi R., Nuzzo A., Origliani G., Modena M.G. (2008). Prognostic role of flow-mediated dilation and cardiac risk factors in post-menopausal women. J. Am. Coll. Cardiol..

[77] Sprung V.S., Atkinson G., Cuthbertson D.J., Pugh C.J., Aziz N., Green D.J., Cable N.T., Jones H. (2013). Endothelial function measured using flow-mediated dilation in polycystic ovary syndrome: A meta-analysis of the observational studies. Clin. Endocrinol.

[78] Gulanski B.I., Flannery C.A., Peter P.R., Leone C.A., Stachenfeld N.S. (2020). Compromised endothelial function in transgender men taking testosterone. Clin. Endocrinol.

[79] Kelly C.J., Speirs A., Gould G.W., Petrie J.R., Lyall H., Connell J.M. (2002). Altered vascular function in young women with polycystic ovary syndrome. J. Clin. Endocrinol. Metab..

[80] Christian R.C., Dumesic D.A., Behrenbeck T., Oberg A.L., Sheedy P.F., Fitzpatrick L.A. (2003). Prevalence and predictors of coronary artery calcification in women with polycystic ovary syndrome. J. Clin. Endocrinol. Metab..

[81] Kim C., Aroda V.R., Goldberg R.B., Younes N., Edelstein S.L., Carrion-Petersen M., Ehrmann D.A., Diabetes Prevention Program Outcomes Study G. (2018). Androgens, Irregular Menses, and Risk of Diabetes and Coronary Artery Calcification in the Diabetes Prevention Program. J. Clin. Endocrinol. Metab..

[82] Meun C., Franco O.H., Dhana K., Jaspers L., Muka T., Louwers Y., Ikram M.A., Fauser B., Kavousi M., Laven J.S.E. (2018). High Androgens in Postmenopausal Women and the Risk for Atherosclerosis and Cardiovascular Disease: The Rotterdam Study. J. Clin. Endocrinol. Metab..

[83] Patel S.S., Truong U., King M., Ferland A., Moreau K.L., Dorosz J., Hokanson J.E., Wang H., Kinney G.L., Maahs D.M. (2017). Obese adolescents with polycystic ovarian syndrome have elevated cardiovascular disease risk markers. Vasc. Med..

[84] Aksun S., Sonu N.C., Aygun S., Karakulak U.N., Mumusoglu S., Yildiz B.O. (2024). Alterations of cardiometabolic risk profile in polycystic ovary syndrome: 13 years follow-up in an unselected population. J. Endocrinol. Invest..

[85] Cooney L.G., Dokras A. (2018). Beyond fertility: Polycystic ovary syndrome and long-term health. Fertil. Steril..

[86] Zhang J., Xu J.H., Qu Q.Q., Zhong G.Q. (2020). Risk of Cardiovascular and Cerebrovascular Events in Polycystic Ovarian Syndrome Women: A Meta-Analysis of Cohort Studies. Front. Cardiovasc. Med..

[87] Ding D.C., Tsai I.J., Wang J.H., Lin S.Z., Sung F.C. (2018). Coronary artery disease risk in young women with polycystic ovary syndrome. Oncotarget.

[88] Prelevic G.M., Beljic T., Balint-Peric L., Ginsburg J. (1995). Cardiac flow velocity in women with the polycystic ovary syndrome. Clin. Endocrinol.

[89] Yarali H., Yildirir A., Aybar F., Kabakci G., Bukulmez O., Akgul E., Oto A. (2001). Diastolic dysfunction and increased serum homocysteine concentrations may contribute to increased cardiovascular risk in patients with polycystic ovary syndrome. Fertil. Steril..

[90] Tay C.T., Mousa A., Vyas A., Pattuwage L., Tehrani F.R., Teede H. (2024). 2023 International Evidence-Based Polycystic Ovary Syndrome Guideline Update: Insights From a Systematic Review and Meta-Analysis on Elevated Clinical Cardiovascular Disease in Polycystic Ovary Syndrome. J. Am. Heart Assoc..

[91] Barrea L., Arnone A., Annunziata G., Muscogiuri G., Laudisio D., Salzano C., Pugliese G., Colao A., Savastano S. (2019). Adherence to the Mediterranean Diet, Dietary Patterns and Body Composition in Women with Polycystic Ovary Syndrome (PCOS). Nutrients.

[92] Szczuko M., Kikut J., Szczuko U., Szydlowska I., Nawrocka-Rutkowska J., Zietek M., Verbanac D., Saso L. (2021). Nutrition Strategy and Life Style in Polycystic Ovary Syndrome-Narrative Review. Nutrients.

[93] Szczuko M., Sankowska P., Zapalowska-Chwyc M., Wysokinski P. (2017). Studies on the quality nutrition in women with polycystic ovary syndrome (PCOS). Rocz. Panstw. Zakl. Hig..

[94] Feng Y., Qi J., Xue X., Li X., Liao Y., Sun Y., Tao Y., Yin H., Liu W., Li S. (2022). Follicular free fatty acid metabolic signatures and their effects on oocyte competence in non-obese PCOS patients. Reproduction.

[95] Khan K.A., Stas S., Kurukulasuriya L.R. (2006). Polycystic ovarian syndrome. J. Cardiometab Syndr..

[96] Rao V.S., Armour M., Patwardhan K., Cheema B.S., Smith C., Sharma R., Ee C. (2023). A Scoping Review of Ayurveda Studies in Women with Polycystic Ovary Syndrome. J. Integr. Complement. Med..

[97] Kaminska W., Wisniewska K., Okreglicka K., Pazura I., Nitsch-Osuch A. (2023). Lifestyle intervention towards Mediterranean Diet, physical activity adherence and anthropometric parameters in normal weight women with Polycystic Ovary Syndrome or Hashimoto’s Thyroiditis—Preliminary study. Ann. Agric. Environ. Med..

[98] Che X., Chen Z., Liu M., Mo Z. (2021). Dietary Interventions: A Promising Treatment for Polycystic Ovary Syndrome. Ann. Nutr. Metab..

[99] Szmidt M.K., Granda D., Madej D., Sicinska E., Kaluza J. (2023). Adherence to the Mediterranean Diet in Women and Reproductive Health across the Lifespan: A Narrative Review. Nutrients.

[100] Barrea L., Muscogiuri G., Pugliese G., de Alteriis G., Colao A., Savastano S. (2021). Metabolically Healthy Obesity (MHO) vs. Metabolically Unhealthy Obesity (MUO) Phenotypes in PCOS: Association with Endocrine-Metabolic Profile, Adherence to the Mediterranean Diet, and Body Composition. Nutrients.

[101] Cincione R.I., Losavio F., Ciolli F., Valenzano A., Cibelli G., Messina G., Polito R. (2021). Effects of Mixed of a Ketogenic Diet in Overweight and Obese Women with Polycystic Ovary Syndrome. Int. J. Environ. Res. Public Health.

[102] Cincione I.R., Graziadio C., Marino F., Vetrani C., Losavio F., Savastano S., Colao A., Laudisio D. (2023). Short-time effects of ketogenic diet or modestly hypocaloric Mediterranean diet on overweight and obese women with polycystic ovary syndrome. J. Endocrinol. Invest..

[103] Paoli A., Mancin L., Giacona M.C., Bianco A., Caprio M. (2020). Effects of a ketogenic diet in overweight women with polycystic ovary syndrome. J. Transl. Med..

[104] Barrea L., Verde L., Camajani E., Cernea S., Frias-Toral E., Lamabadusuriya D., Ceriani F., Savastano S., Colao A., Muscogiuri G. (2023). Ketogenic Diet as Medical Prescription in Women with Polycystic Ovary Syndrome (PCOS). Curr. Nutr. Rep..

[105] Singh S., Pal N., Shubham S., Sarma D.K., Verma V., Marotta F., Kumar M. (2023). Polycystic Ovary Syndrome: Etiology, Current Management, and Future Therapeutics. J. Clin. Med..

[106] Magagnini M.C., Condorelli R.A., Cimino L., Cannarella R., Aversa A., Calogero A.E., La Vignera S. (2022). Does the Ketogenic Diet. Improve the Quality of Ovarian Function in Obese Women?. Nutrients.

[107] Brennan L., Teede H., Skouteris H., Linardon J., Hill B., Moran L. (2017). Lifestyle and Behavioral Management of Polycystic Ovary Syndrome. J. Womens Health.

[108] Ehrmann D.A. (2005). Polycystic ovary syndrome. N. Engl. J. Med..

[109] Stepto N.K., Cassar S., Joham A.E., Hutchison S.K., Harrison C.L., Goldstein R.F., Teede H.J. (2013). Women with polycystic ovary syndrome have intrinsic insulin resistance on euglycaemic-hyperinsulaemic clamp. Hum. Reprod..

[110] Cameron A.R., Morrison V.L., Levin D., Mohan M., Forteath C., Beall C., McNeilly A.D., Balfour D.J., Savinko T., Wong A.K. (2016). Anti-Inflammatory Effects of Metformin Irrespective of Diabetes Status. Circ. Res..

[111] Xu X., Du C., Zheng Q., Peng L., Sun Y. (2014). Effect of metformin on serum interleukin-6 levels in polycystic ovary syndrome: A systematic review. BMC Womens Health.

[112] Zeng X., Xie Y.J., Liu Y.T., Long S.L., Mo Z.C. (2020). Polycystic ovarian syndrome: Correlation between hyperandrogenism, insulin resistance and obesity. Clin. Chim. Acta.

[113] Dunaif A. (2008). Drug insight: Insulin-sensitizing drugs in the treatment of polycystic ovary syndrome--a reappraisal. Nat. Clin. Pract. Endocrinol. Metab..

[114] Dumitrescu R., Mehedintu C., Briceag I., Purcarea V.L., Hudita D. (2015). Metformin-clinical pharmacology in PCOs. J. Med. Life.

[115] Escobar-Morreale H.F., Carmina E., Dewailly D., Gambineri A., Kelestimur F., Moghetti P., Pugeat M., Qiao J., Wijeyaratne C.N., Witchel S.F. (2012). Epidemiology, diagnosis and management of hirsutism: A consensus statement by the Androgen Excess and Polycystic Ovary Syndrome Society. Hum. Reprod. Update.

[116] Guan Y., Wang D., Bu H., Zhao T., Wang H. (2020). The Effect of Metformin on Polycystic Ovary Syndrome in Overweight Women: A Systematic Review and Meta-Analysis of Randomized Controlled Trials. Int. J. Endocrinol..

[117] Ramachandran A., Snehalatha C., Mary S., Mukesh B., Bhaskar A.D., Vijay V., Indian Diabetes Prevention P. (2006). The Indian Diabetes Prevention Programme shows that lifestyle modification and metformin prevent type 2 diabetes in Asian Indian subjects with impaired glucose tolerance (IDPP-1). Diabetologia.

[118] Otaghi M., Azami M., Khorshidi A., Borji M., Tardeh Z. (2019). The association between metabolic syndrome and polycystic ovary syndrome: A systematic review and meta-analysis. Diabetes Metab. Syndr..

[119] Oikonomou E., Xenou M., Zakynthinos G.E., Tsaplaris P., Lampsas S., Bletsa E., Gialamas I., Kalogeras K., Goliopoulou A., Gounaridi M.I. (2023). Novel Approaches to the Management of Diabetes Mellitus in Patients with Coronary Artery Disease. Curr. Pharm. Des..

[120] Dokras A., Sarwer D.B., Allison K.C., Milman L., Kris-Etherton P.M., Kunselman A.R., Stetter C.M., Williams N.I., Gnatuk C.L., Estes S.J. (2016). Weight Loss and Lowering Androgens Predict Improvements in Health-Related Quality of Life in Women with PCOS. J. Clin. Endocrinol. Metab..

[121] Clark A.M., Thornley B., Tomlinson L., Galletley C., Norman R.J. (1998). Weight loss in obese infertile women results in improvement in reproductive outcome for all forms of fertility treatment. Hum. Reprod..

[122] Mutsaerts M.A., van Oers A.M., Groen H., Burggraaff J.M., Kuchenbecker W.K., Perquin D.A., Koks C.A., van Golde R., Kaaijk E.M., Schierbeek J.M. (2016). Randomized Trial of a Lifestyle Program in Obese Infertile Women. N. Engl. J. Med..

[123] ACOG (2015). Practice Bulletin No 156: Obesity in Pregnancy. Obstet. Gynecol..

[124] Szczesnowicz A., Szeliga A., Niwczyk O., Bala G., Meczekalski B. (2023). Do GLP-1 Analogs Have a Place in the Treatment of PCOS? New Insights and Promising Therapies. J. Clin. Med..

[125] Nauck M.A., Quast D.R., Wefers J., Meier J.J. (2021). GLP-1 receptor agonists in the treatment of type 2 diabetes—State-of-the-art. Mol. Metab..

[126] Holst J.J. (2007). The physiology of glucagon-like peptide. Physiol. Rev..

[127] Aldawsari M., Almadani F.A., Almuhammadi N., Algabsani S., Alamro Y., Aldhwayan M. (2023). The Efficacy of GLP-1 Analogues on Appetite Parameters, Gastric Emptying, Food Preference and Taste Among Adults with Obesity: Systematic Review of Randomized Controlled Trials. Diabetes Metab. Syndr. Obes..

[128] Ikonomidis I., Pavlidis G., Thymis J., Birba D., Kalogeris A., Kousathana F., Kountouri A., Balampanis K., Parissis J., Andreadou I. (2020). Effects of Glucagon-Like Peptide-1 Receptor Agonists, Sodium-Glucose Cotransporter-2 Inhibitors, and Their Combination on Endothelial Glycocalyx, Arterial Function, and Myocardial Work Index in Patients with Type 2 Diabetes Mellitus after 12-Month Treatment. J. Am. Heart Assoc..

[129] Katogiannis K., Thymis J., Kousathana F., Pavlidis G., Korakas E., Kountouri A., Balampanis K., Prentza V., Kostelli G., Michalopoulou H. (2024). Effects of Liraglutide, Empagliflozin and Their Combination on Left Atrial Strain and Arterial Function. Medicina.

[130] Tsigkou V., Oikonomou E., Anastasiou A., Lampsas S., Zakynthinos G.E., Kalogeras K., Katsioupa M., Kapsali M., Kourampi I., Pesiridis T. (2023). Molecular Mechanisms and Therapeutic Implications of Endothelial Dysfunction in Patients with Heart Failure. Int. J. Mol. Sci..

[131] Frossing S., Nylander M., Chabanova E., Frystyk J., Holst J.J., Kistorp C., Skouby S.O., Faber J. (2018). Effect of liraglutide on ectopic fat in polycystic ovary syndrome: A randomized clinical trial. Diabetes Obes. Metab..

[132] Nylander M., Frossing S., Clausen H.V., Kistorp C., Faber J., Skouby S.O. (2017). Effects of liraglutide on ovarian dysfunction in polycystic ovary syndrome: A randomized clinical trial. Reprod. Biomed. Online.

[133] Han Y., Li Y., He B. (2019). GLP-1 receptor agonists versus metformin in PCOS: A systematic review and meta-analysis. Reprod. Biomed. Online.

[134] Ge J.J., Wang D.J., Song W., Shen S.M., Ge W.H. (2022). The effectiveness and safety of liraglutide in treating overweight/obese patients with polycystic ovary syndrome: A meta-analysis. J. Endocrinol. Invest..

[135] Zhou L., Qu H., Yang L., Shou L. (2023). Effects of GLP1RAs on pregnancy rate and menstrual cyclicity in women with polycystic ovary syndrome: A meta-analysis and systematic review. BMC Endocr. Disord..

[136] Siamashvili M., Davis S.N. (2021). Update on the effects of GLP-1 receptor agonists for the treatment of polycystic ovary syndrome. Expert. Rev. Clin. Pharmacol..

[137] Martin K.A., Anderson R.R., Chang R.J., Ehrmann D.A., Lobo R.A., Murad M.H., Pugeat M.M., Rosenfield R.L. (2018). Evaluation and Treatment of Hirsutism in Premenopausal Women: An Endocrine Society Clinical Practice Guideline. J. Clin. Endocrinol. Metab..

[138] Messinis I.E. (2006). Ovarian feedback, mechanism of action and possible clinical implications. Hum. Reprod. Update.

[139] Choi J., Smitz J. (2014). Luteinizing hormone and human chorionic gonadotropin: Distinguishing unique physiologic roles. Gynecol. Endocrinol..

[140] Rashid R., Mir S.A., Kareem O., Ali T., Ara R., Malik A., Amin F., Bader G.N. (2022). Polycystic ovarian syndrome-current pharmacotherapy and clinical implications. Taiwan. J. Obstet. Gynecol..

[141] Kitzinger C., Willmott J. (2002). ‘The thief of womanhood’: Women’s experience of polycystic ovarian syndrome. Soc. Sci. Med..

[142] Melin J., Forslund M., Alesi S., Piltonen T., Romualdi D., Spritzer P.M., Tay C.T., Pena A., Witchel S.F., Mousa A. (2024). Metformin and Combined Oral Contraceptive Pills in the Management of Polycystic Ovary Syndrome: A Systematic Review and Meta-analysis. J. Clin. Endocrinol. Metab..

[143] Wu L., Liu Y., Huang X., Lin K., Liu Y., Li Z., Wei T., Song L., Hua Y., Wang X. (2023). Oral contraceptives (OCs) in combination with metformin versus OCs alone on metabolism in nonobese polycystic ovary syndrome: A meta-analysis and systematic review of randomized controlled trials. Clin. Endocrinol.

[144] Milewska E.M., Czyzyk A., Meczekalski B., Genazzani A.D. (2016). Inositol and human reproduction. From cellular metabolism to clinical use. Gynecol. Endocrinol..

[145] Facchinetti F., Unfer V., Dewailly D., Kamenov Z.A., Diamanti-Kandarakis E., Lagana A.S., Nestler J.E., Soulage C.O., for the Group of ‘Inositol in PCOS and Reproduction’ (2020). Inositols in Polycystic Ovary Syndrome: An Overview on the Advances. Trends Endocrinol. Metab..

[146] Dinicola S., Unfer V., Facchinetti F., Soulage C.O., Greene N.D., Bizzarri M., Lagana A.S., Chan S.Y., Bevilacqua A., Pkhaladze L. (2021). Inositols: From Established Knowledge to Novel Approaches. Int. J. Mol. Sci..

[147] Baillargeon J.P., Nestler J.E., Ostlund R.E., Apridonidze T., Diamanti-Kandarakis E. (2008). Greek hyperinsulinemic women, with or without polycystic ovary syndrome, display altered inositols metabolism. Hum. Reprod..

[148] Pundir J., Psaroudakis D., Savnur P., Bhide P., Sabatini L., Teede H., Coomarasamy A., Thangaratinam S. (2018). Inositol treatment of anovulation in women with polycystic ovary syndrome: A meta-analysis of randomised trials. BJOG Int. J. Obstet. Gynaecol..

[149] Unfer V., Facchinetti F., Orru B., Giordani B., Nestler J. (2017). Myo-inositol effects in women with PCOS: A meta-analysis of randomized controlled trials. Endocr. Connect..

[150] Zeng L., Yang K. (2018). Effectiveness of myoinositol for polycystic ovary syndrome: A systematic review and meta-analysis. Endocrine.

[151] Dona G., Sabbadin C., Fiore C., Bragadin M., Giorgino F.L., Ragazzi E., Clari G., Bordin L., Armanini D. (2012). Inositol administration reduces oxidative stress in erythrocytes of patients with polycystic ovary syndrome. Eur. J. Endocrinol..

[152] Fruzzetti F., Perini D., Russo M., Bucci F., Gadducci A. (2017). Comparison of two insulin sensitizers, metformin and myo-inositol, in women with polycystic ovary syndrome (PCOS). Gynecol. Endocrinol..

[153] Fatima K., Jamil Z., Faheem S., Adnan A., Javaid S.S., Naeem H., Mohiuddin N., Sajid A., Ochani S. (2023). Effects of myo-inositol vs. metformin on hormonal and metabolic parameters in women with PCOS: A meta-analysis. Ir. J. Med. Sci..

[154] McCartney C.R., Marshall J.C. (2016). CLINICAL PRACTICE. Polycystic Ovary Syndrome. N. Engl. J. Med..

[155] Eisenberg D., Shikora S.A., Aarts E., Aminian A., Angrisani L., Cohen R.V., De Luca M., Faria S.L., Goodpaster K.P.S., Haddad A. (2022). 2022 American Society for Metabolic and Bariatric Surgery (ASMBS) and International Federation for the Surgery of Obesity and Metabolic Disorders (IFSO): Indications for Metabolic and Bariatric Surgery. Surg Obes. Relat. Dis..

[156] Hu L., Ma L., Xia X., Ying T., Zhou M., Zou S., Yu H., Yin J. (2022). Efficacy of Bariatric Surgery in the Treatment of Women with Obesity and Polycystic Ovary Syndrome. J. Clin. Endocrinol. Metab..

[157] Holte J., Bergh T., Berne C., Wide L., Lithell H. (1995). Restored insulin sensitivity but persistently increased early insulin secretion after weight loss in obese women with polycystic ovary syndrome. J. Clin. Endocrinol. Metab..

[158] Skubleny D., Switzer N.J., Gill R.S., Dykstra M., Shi X., Sagle M.A., de Gara C., Birch D.W., Karmali S. (2016). The Impact of Bariatric Surgery on Polycystic Ovary Syndrome: A Systematic Review and Meta-analysis. Obes. Surg..

[159] Li Y.J., Han Y., He B. (2019). Effects of bariatric surgery on obese polycystic ovary syndrome: A systematic review and meta-analysis. Surg. Obes. Relat. Dis..

[160] Bhandari M., Kosta S., Bhandari M., Reddy M., Mathur W., Gupta M. (2022). Effects of Bariatric Surgery on People with Obesity and Polycystic Ovary Syndrome: A Large Single Center Study from India. Obes. Surg..

